# Addressing pollination deficits in orchard crops through habitat management for wild pollinators

**DOI:** 10.1002/eap.2743

**Published:** 2022-11-30

**Authors:** Michael P. D. Garratt, Rory S. O'Connor, Claire Carvell, Michelle T. Fountain, Tom D. Breeze, Richard Pywell, John W. Redhead, Lois Kinneen, Nadine Mitschunas, Louise Truslove, Celina Xavier e Silva, Nigel Jenner, Caroline Ashdown, Claire Brittain, Megan McKerchar, Charnee Butcher, Mike Edwards, Marek Nowakowski, Peter Sutton, Simon G. Potts

**Affiliations:** ^1^ Centre for Agri‐Environmental Research, University of Reading Reading UK; ^2^ UK Centre for Ecology & Hydrology Wallingford UK; ^3^ NIAB East Malling Kent UK; ^4^ Avalon Fresh Limited, The Apple Shed Maidstone UK; ^5^ Avalon Growers Producer Organisation Ltd Louth Lincolnshire UK; ^6^ Syngenta, Jealotts Hill International Research Centre Bracknell UK; ^7^ Worldwide Fruit Limited, Apple Way Spalding UK; ^8^ Edwards Ecological and Data Services Ltd Midhurst UK; ^9^ Wildlife Farming Company Chesterton Oxfordshire UK

**Keywords:** apples, bumblebee, floral resources, honeybee, hoverfly, nesting, pollinator, solitary bee

## Abstract

There is increasing evidence that farmers in many areas are achieving below maximum yields due to insufficient pollination. Practical and effective approaches are needed to maintain wild pollinator populations within agroecosystems so they can deliver critical pollination services that underpin crop production. We established nesting and wildflower habitat interventions in 24 UK apple orchards and measured effects on flower‐visiting insects and the pollination they provide, exploring how this was affected by landscape context. We quantified the extent of pollination deficits and assessed whether the management of wild pollinators can reduce deficits and deliver improved outcomes for growers over 3 years. Wildflower interventions increased solitary bee numbers visiting apple flowers by over 20%, but there was no effect of nesting interventions. Other pollinator groups were influenced by both local and landscape‐scale factors, with bumblebees and hoverflies responding to the relative proportion of semi‐natural habitat at larger spatial scales (1000 m), while honeybees and other flies responded at 500 m or less. By improving fruit number and quality, pollinators contributed more than £16 k per hectare. However, deficits (where maximum potential was not being reached due to a lack of pollination) were recorded and the extent of these varied across orchards, and from year to year, with a 22% deficit in output in the worst (equivalent to ~£14 k/ha) compared to less than 3% (equivalent to ~£2 k/ha) in the best year. Although no direct effect of our habitat interventions on deficits in gross output was observed, initial fruit set and seed set deficits were reduced by abundant bumblebees, and orchards with a greater abundance of solitary bees saw lower deficits in fruit size. The abundance of pollinators in apple orchards is influenced by different local and landscape factors that interact and vary between years. Consequently, pollination, and the extent of economic output deficits, also vary between orchards and years. We highlight how approaches, including establishing wildflower areas and optimizing the ratio of cropped and non‐cropped habitats can increase the abundance of key apple pollinators and improve outcomes for growers.

## INTRODUCTION

The role of wild insect pollinators as significant contributors to crop production globally is widely recognized (Dainese et al., 2019). Yet evidence for global and local pollinator declines is building (Potts et al., 2016; Powney et al., 2019). Combined with increasing demand for insect pollinated crops (Aizen et al., 2019), this risk of decline threatens ecologically and economically valuable pollination services. Habitat loss from agricultural expansion is a key driver of losses in wild pollinator abundance and diversity, in turn threatening pollination services to crops (Dainese et al., 2019; Gardner et al., 2021; Martin et al., 2019). Deficits in crop yield due to a lack of pollination service are apparent (Garibaldi et al., 2016; Reilly et al., 2020); therefore, practical and effective management tools that protect pollination services in agro‐ecosystems are urgently needed (Garibaldi et al., 2019; Kleijn et al., 2019).

Apples (*Malus domestica*) are a globally important crop with a high sale price and high dependence on insects for pollination (Garratt et al., 2014; Samnegård, Hambäck, & Smith, 2019). Depending on where apples are grown, they are pollinated by different pollinator groups include bumblebees, other wild bees, and hoverflies (Blitzer et al., 2016; Földesi et al., 2016; Pardo & Borges, 2020), and, in some situations, growers rely on honeybees (Rollin & Garibaldi, 2019; Stern et al., 2001). There is growing evidence that the abundance and diversity of flower visiting insects to apples can be influenced by landscape context (Bartholomée et al., 2020; Földesi et al., 2016; Joshi et al., 2016; Martins et al., 2015) and farm management (Samnegård, Alins, et al., 2019) due to their effects on the availability of resources such as alternative forage and nesting habitat. Provision of local floral resources can typically increase (Samnegård, Alins, et al., 2019), or rarely decrease (Osterman et al., 2021) visitors to apple blossoms. However, the extent to which the availability of nesting sites limits populations of apple pollinators is less well understood (Antoine & Forrest, 2021; Harmon‐Threatt, 2020).

In UK orchards, apple blossoms are visited by managed honeybees, wild bumblebees, and solitary bees, as well as hoverflies and other insects. However, ground‐nesting solitary bees (e.g. *Andrena* sp.) have been identified as especially important contributors to pollination (Garratt et al., 2016; Hutchinson et al., 2021). Ground‐nesting bee species nest in areas of undisturbed soil, often south facing with low vegetation cover (Antoine & Forrest, 2021; Harmon‐Threatt, 2020), such potential nesting areas exist in orchards between vegetated alleyways and at the end of trees rows but the extent to which availability of these habitats constrain populations is not known. Floral resources have been introduced into apple orchards as flowering plants in between apple rows (Campbell et al., 2017; McKerchar et al., 2020) or adjacent to orchards (Carvell et al., 2021; Heller et al., 2019) to promote pollinators. Such areas are utilized as a pollen and nectar resource by wild pollinators (Carvell et al., 2021; Heller et al., 2019), but whether these habitats are sufficient to support populations of important taxa with resulting improved crop pollination and how this is affected by existing landscape context is unknown.

There is considerable policy support and economic incentives for agri‐environmental schemes that offer subsidies for habitat management for species conservation. However, by protecting species richness and diversity such approaches can help deliver pollination for more sustainable production of crops within the agroecosystem (Garibaldi et al., 2019). There is a need to promote greater, more resilient pollination services and crop production in current farming contexts by using management practices to “ecologically intensify” production (Kleijn et al., 2019), irrespective of additional economic incentives through agri‐environment schemes. Deficits in apple production due to insufficient pollination, particularly through sub‐optimal fruit quality, have been identified (Garratt et al., 2014; Samnegård, Hambäck, & Smith, 2019). This provides a clear opportunity to deliver better outcomes for growers through improved management of wild pollinators (Garratt et al., 2021). Practical management approaches that promote pollination by key pollinators are needed, including an understanding of how their effectiveness is influenced by existing habitats or wider landscape context.

This study tested whether wildflower and nesting habitat interventions influence pollinators visiting apple blossoms and the pollination they provide. Specifically, the objectives were to: (i) test effects of targeted nesting and wildflower habitat interventions on known apple pollinators and understand how these are modified by existing habitats; (ii) quantify the agronomic and economic extent of yield and quality deficits in apple orchards; and (iii) explore whether wild pollinator management can reduce deficits to support improved production outcomes for growers.

## MATERIALS AND METHODS

### Study design

This study involved 24 conventionally managed commercial apple (*Malus domestica*) orchards (cv. Gala apple) in Kent, UK. In 2016, orchards were assigned to one of four treatments: (1) a wildflower intervention, (2) a nesting intervention, (3) both a wildflower and nesting intervention, and (4) control orchards, receiving no pollinator management intervention. Otherwise, orchards received standard conventional management as determined by individual growers. A 40 m by 50 m study plot was established along one edge of each orchard from which pollinator and pollination assessments were made (Appendix S1: Figure S1). Orchards ranged in size from 0.47 to 6.43 ha (mean = 2.05 ha) with a minimum separation distance of 300 m between the edges of study plots in each orchard. Orchards were assigned into six study blocks of four orchards grouped by spatial proximity, with each of the four treatments within each block. During surveys, orchards were sampled in their study block, and the order in which they were visited was randomized for each survey round. One wildflower area did not establish, and the orchard was excluded leaving 23 orchards across six blocks (Appendix S1: Figure S1). Two orchards used in 2017 (one nesting intervention and one control orchard) were unavailable in 2018 and were replaced by different orchards meeting our study criteria.

### Habitat interventions

#### Wildflower habitat intervention

Wildflower areas were established adjacent to each of the 11 study orchards receiving the wildflower intervention. To standardize the size relative to the cover of orchards, wildflower areas were established to cover between 1% and 3% of the total area of orchards within a radius of 500 m around the orchard, resulting in plots ranging from 0.07 to 0.62 ha. Seed beds were lightly harrowed and treated with herbicide prior to sowing in autumn 2016 or spring 2017, depending on weed pressure and soil conditions. During the first year, wildflower areas were cut in June or July and again in October, and for the remainder of the study cutting took place in September. The seed mix was designed to provide a range of flower morphologies to encourage utilization by the diversity of wild pollinators of UK apples (Garratt et al., 2016) and to maximize provision of pollen and nectar throughout the period after apple flowering (UK summertime). As such, 15 perennial wildflowers and four low‐growing fine grass species were selected (Appendix S1: Table S1). In addition, to provide floral resources for pollinators during the first year of the study (2017), 10 annual species were over‐sown to cover an area of between 100 and 200 m^2^ depending on size of the wildflower area. Further details are in Carvell et al., 2021.

#### Bee nesting habitat intervention

Research has shown that ground‐nesting Andrenid bees are important pollinators of apples (Garratt et al., 2016; Pardo & Borges, 2020). These species are often found nesting in bare soils with good sun exposure (Antoine & Forrest, 2021). To increase the availability of potential nest sites for ground nesting bees we extended the herbicide spray areas along the full length of the orchard edge adjacent to our study plots in the 12 orchards receiving the nesting intervention. Glyphosate was applied in spring 2017 at 5 L/ha extending the area of herbicide strip beyond the tree row so that the existing area of bare ground was doubled resulting in between 2.2 and 3.3 m^2^ additional bare ground per row. Excess vegetation was removed from the plots by raking the surface, aiming for the nesting areas to be 80% clear of vegetation from before apple blossom (April) to the middle of the summer (July). The herbicide treatment was re‐applied before blossom each year (Appendix S1: Figure S2).

### Existing floral and nesting resources

Locally available existing spring floral resources were assessed in each study orchard. Floral cover of all non‐crop habitats within, and immediately around each study orchard, were measured in May of each year. Each habitat parcel inside and within a 5 m buffer of the orchard was mapped (including headlands, margins, and hedgerows) to quantify its area for each orchard. Floral surveys were carried out to establish percentage cover of key spring‐flowering non‐crop forage plant species or species groups within each parcel and the percentage in flower. Subsequently, the total area of non‐crop flowers in each orchard was estimated as the sum of the area in flower. Key spring‐flowering non‐crop forage plants included *Trifolium repens*, *Cirsium* spp., yellow and white composites for example, *Taraxacum* spp., *Apiaceae* or *Umbelliferae*, and woody hedgerow species (e.g., *Crataegus, Salix, Prunus*, and *Rubus* spp.) (Redhead et al., 2018). As study orchards varied in size, to estimate the relative availability of floral resources, the proportional flower cover of key flowering species or plant groups was used.

To estimate the availability of existing nesting resources within each orchard, the area of bare‐ground in and around each orchard was mapped. The total herbicide sprayed area under trees was estimated by measuring the herbicide tree row width and by considering the total length of tree rows across the orchards. Other bare‐ground areas were quantified during flower surveys, by estimating the percentage of each parcel covered with bare‐ground. These maps were digitized in ArcGIS, the total area of bare‐ground in m^2^ was estimated for each orchard and as for floral resources, to standardize the availability of bare‐ground a relative proportion was calculated.

Semi‐natural habitats in agricultural areas can provide a source of both nesting and forage resources for pollinators (Bartholomée et al., 2020). In addition, mass flowering crops within a landscape can serve as a source of forage, or alternatively can act to dilute the availability of crop pollinators (Holzschuh et al., 2016; Shaw et al., 2020). Therefore, the coverage of all tree fruit orchards surrounding each study orchard was calculated using aerial imagery, and semi‐natural land cover (broadleaved woodland + neutral grassland) was derived from the UK‐CEH Land Cover Map 2015 (Rowland et al., 2017) and OS Vector Map Local (https://www.ordnancesurvey.co.uk/business-government/products/vectormap-local). The ratio of orchards to semi‐natural habitats was calculated at three spatial scales (250, 500 and 1000 m radius buffers from the center of each orchard) because, due to differences in foraging ranges and life histories, different functional groups of pollinator are likely to be affected by landscape context at different scales (Greenleaf et al., 2007).

### Pollinators and pollination

Within each study plot, 50 m transects were marked out on three centrally located tree rows spaced at least 10 m apart (Appendix S1: Figure S1). During apple flowering for the 3 years of the study, pollinator surveys were carried out by walking each transect over a 20‐min period, recording all flower‐visiting insects on apple blossoms in a 1 m wide moving observation area, down one side of the tree row. Pollinator surveys were done between 10:00 AM and 4:00 PM and only when whether conditions exceeded a minimum threshold of 17°C on cloudy days and wind speeds were below 4 on the Beaufort scale. A minimum temperature threshold of 13–17°C was used in sunny conditions. Pollinators were recorded to broad functional groups including honeybees, bumblebees, solitary bees (i.e., any other wild bee), hoverflies, and other flies. The number of open flowers on the transect was also estimated by counting the number of flower clusters on three trees per transect and the number of flowers per cluster on 10 clusters on each tree. By multiplying the mean flowers per cluster by the number of clusters per tree and the number of trees along the transect, a total flower number was calculated. At least three survey rounds were carried out per orchard per year.

To measure the contribution of insect pollinators to apple yield and quality and to assess the extent of any pollination deficits, 5 evenly spaced trees along each of the study transects (15 trees per orchard) were selected at the start of the study. Prior to bloom, three branches on each tree were randomly assigned one of three pollination treatments. One involved the exclusion of insect pollinators using a PVC mesh bag with 1 mm aperture size placed over the branch just prior to bloom and removed at the end of bloom (hereafter “exclusion” treatment). The second treatment was an open control where branches were left unmanipulated and insects could visit the blossoms as normal (hereafter “open” treatment). The third treatment was a supplementary pollination treatment where pollen was collected from polliniser trees within each orchard and applied using a paint brush to open flowers on the supplementary pollinated branch. This was done during each visit to the orchard during the bloom period (hereafter the “supplementary” treatment).

On each study tree the proportion of flowers which set as fruitlets in June (Early fruit set) and the proportion of flowers which remained as fruit at harvest (Final fruit set) was recorded. One apple was collected from each branch and assessed for maximum width in cm using calipers to the nearest mm, fruit weight measured on an electric balance, and the number of seeds per apple. The fruit were scored for shape using a four‐point scale (1 = regular shaped, 2 = slight shape irregularity, 3 = moderate shape irregularity, 4 = severe shape irregularity). For each treatment apple gross output, the monetary sale value of apples produced by the branch, was calculated considering fruit weight and the proportion of class 1 and class 2 fruit and the price of these fruits. Class 1 fruit are those that achieve a minimum size and shape threshold to command an improved price for growers and information on these was provided by our industry partners. We used gross output per branch as a metric of pollination as this captures the full extent of both yield and quality parameters in a single metric that is relevant to producers. Gross output was calculated as follows:

$$\begin{array}{ll} {\mathrm{GO}}_{\mathrm{xbt}} & =\left({\mathrm{ffs}}_{\mathrm{xbt}}\times {\mathrm{fw}}_{\mathrm{xbt}}\times \mathrm{PC}1_{\mathrm{xbt}}\times \text{£}C1\right)\\ & \;+\left({\mathrm{ffs}}_{\mathrm{xbt}}\times {\mathrm{fw}}_{\mathrm{xbt}}\times \left(1-\mathrm{PC}1_{\mathrm{xbt}}\right)\times \text{£}C2\right) \end{array}$$

where *GO*_*xbt*_ is the gross (pre‐cost) economic output in treatment *x* (open, excluded or supplementary), on branch *b* in year *t*; *ffs* is the final fruit set under treatment *x*, *fw* is the fresh weight of apples harvested, *PC*1 is the proportion of class 1 fruit. *£C*1 is the average price of class 1 fruit per kilo reported by Defra between 2016 and 2017 (Defra, 2022), and *£C*2 is the average price for class 2 fruit.

### Statistical analysis

#### Habitat interventions and apple pollinators

Mixed effects models were used to explore the effects of habitat interventions on the abundance of insect visitors to apple blossom and how this was affected by existing floral and nesting resources as well as the relative availability of semi‐natural habitats at larger scales. Habitat intervention, proportion of key forage (or proportion of bare‐ground for the nesting only models), semi‐natural habitat to orchard ratio, year, and all possible two‐way interactions were included as fixed variables. Total flower number on transects was included as a covariate. Transect nested within orchard, nested within study block were included as random effects. For solitary bee abundance, an initial model including four levels of habitat intervention (floral, nesting, both, and control) was run. Habitat intervention was retained in the model and a post hoc test showed a near significant difference between wildflower interventions and control orchards only (*p* = 0.0775). Therefore, for solitary bees, interventions were tested individually for flowering and nesting versus their respective controls (e.g., for nesting: nesting + both vs. control + floral). For other pollinator groups (bumblebees, honeybees, hoverflies and other flies), only wildflower interventions were considered (floral + both vs. control + nesting) as bare ground is not considered an important habitat for these groups. Models were run separately for each scale of semi‐natural habitat to orchard ratio (250, 500 and 1000 m), and results are presented for the spatial scale for which the models had the lowest AIC_c_. Pollinator abundance per transect was averaged across survey rounds and log transformed prior to analysis. Candidate models were ranked according to Akaike's Information Criteria (AIC) and all models ≤2 ΔAIC_c_ of the top model were averaged to obtain parameter estimates, *z*‐values and *p*‐values for factors remaining in the models considering “full averages” (Burnham & Anderson, 2002).

Because the effect of landscape context was explored at multiple scales (250, 500, and 1000 m) that were often larger than the separation between individual orchards, we tested pollinator abundance for spatial autocorrelation using a Moran's I test. See Supplementary materials for details. All statistical analysis was carried out in R version 4.0.3. For mixed effects models the package LME4 (Bates et al., 2015) was used, and for model averaging we used the MuMIn package (Barton, 2009).

#### Pollination deficits

To investigate the effects of pollination treatment (excluded, open, supplementary) on apple fruit set and quality characteristics, linear mixed effects models were used. Pollination treatment and year were included as fixed effects with tree nested within transect, within orchard, within block included as random effects. For seed number per apple and fruit set, generalized linear mixed models were used with a Poisson and binomial error structure respectively. Model selection and averaging was carried out as for previous models. To explore pairwise differences between pollination treatments, Tukey post hoc tests were carried out using the Multcomp package (Hothorn et al., 2008).

Deficits in fruit set and quality characteristics (size, apple weight, seed set) were calculated as follows:

$$\text{Deficit}=\frac{\text{OSupp}-\text{OOpen}}{\text{OSupp}}$$

where OSupp is the output under supplementary pollination, OOpen is the output under open pollination. In cases where the output achieved following open pollination conditions was greater than for supplementary pollination, the deficit was calculated as a proportion of the output under open pollination. Thus, the deficit is a proportion of maximum crop output allowing both positive and negative values to account for possible limitations due to insufficient pollination (positive deficit values) but also risks of suboptimal output due to over‐pollination (negative deficit value) causing rising costs and/or lower fruit quality (Garratt et al., 2014).

Effects of habitat interventions on deficits in gross output was assessed using mixed effects models with year, habitat intervention, semi‐natural to orchard ratio, proportion of key forage (in flower plot only models), and proportion of bare‐ground (for the nesting only models) as main effects, and transect, nested within orchard, nested within block, as random effects. The four levels of habitat intervention were tested first, and as they were not retained in the final model, wildflower and nesting interventions were tested separately. To explore the extent of gross output deficits between orchards, 95% confidence intervals were calculated, an orchard was considered to have a significant deficit if confidence intervals did not overlap zero (when open and supplementary pollination are equal and indicative of no pollination deficit).

Finally, to examine the relationship between the abundance of different pollinator groups and deficits in apple fruit set, quality, and gross output, mixed models were used. In these models, each pollinator group and its interaction with year were included as main effects with transect, nested within orchard, nested within block as random effects.

#### Orchard scale economic assessments

To upscale estimates of economic benefit, we use average data across whole orchards. For each orchard, in each year, we calculated (i) the net economic benefits of pollination and (ii) the net economic extent of pollination deficits per hectare. Using the national average yield of Gala orchards for each year (DEFRA, 2022), we estimated the net yield (kg/ha) of class 1 and class 2 apples and then multiplied this by the market price/kg for each class (as described above) to get an estimate of typical mean economic gross output of gala orchards. We then accounted for changes in thinning costs using the median costs of pruning from Redman (2020) and difference in each trees' post‐abortion fruit set from the average open set. This provides a measure of the total economic net output per hectare under each treatment, expressed mathematically as:

$$\begin{array}{ll} \mathrm{Net}O_{\mathrm{xrt}} & =\sum_{n=1}^{\mathrm{PC}}({\mathrm{NY}}_{t}\times \left(\frac{{\mathrm{FFS}}_{\mathrm{xrt}}}{\text{mean}\;{\mathrm{FFS}}_{\text{open},t}}\right)\\ & \;\times \left({\mathrm{PC}}_{\text{nxrt}}\times \frac{W_{xnrt}}{W_{open,nrt}}\times \text{£}C_{n}\right))-\left(NT\times T_{xnr}\right) \end{array}$$

where Net*O*_*xrt*_ is the net economic output (£/ha) in treatment *x* (open, excluded, or supplementary) in orchard *r* in year *t*, NY_*t*_ is the annual national average gala yield, FFS_*xrt*_ is the final fruit set, mean FFS_open,*t*_ is the mean final fruit set from all study orchards, PC_*nxrt*_ is the percentage of apples in class *n*, W_*xnrt*_ is the weight of apples in class *n*, £*C*_*n*_ is the price/kg of apples of class *n* (average of 2016 and 2017, DEFRA, 2022), EFS_*xrt*_ is early fruit set, mean EFS_open,*t*_ is the mean early fruit set from all study orchards, and *T* is the median thinning cost (constant between years). We use net output in order to capture the full, orchard scale effects of output variation on relative profitability. The difference between the open and excluded net output (£/ha) in each orchard, in each year, reflects the economic benefits of pollination. The difference between the supplementary and open treatments represents the economic loss due to deficit (or risk of loss due to over‐pollination where deficits are negative).

## RESULTS

### Habitat interventions and apple pollinators

**FIGURE 1:**
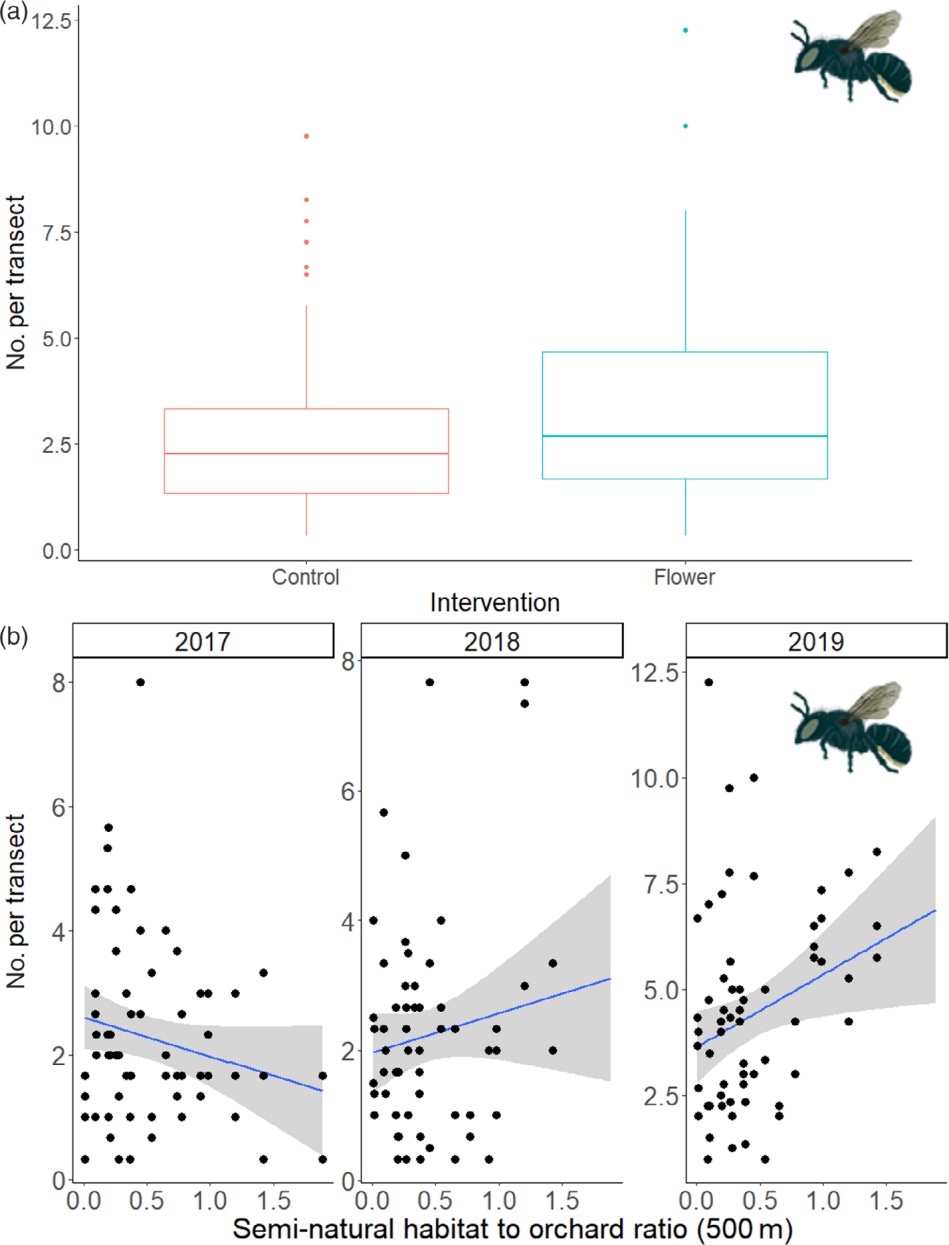
Relationships between solitary bee abundance on apple blossoms and (a) wildflower habitat intervention (*n* = 11) versus control orchards (*n* = 12), and (b) semi‐natural habitat to orchard ratio at a 500 m radius. Data for 23 apple orchards over 3 years included (linear model with 95% confidence intervals shown). Mean abundance of solitary bees was significantly different between wildflower habitat interventions and controls according to a linear mixed effects model (*z* = 2.351, *p* = 0.019).

Over the 23 orchards and 3 years of the study, we recorded 1262 bumblebees, 5820 honeybees, 2031 solitary bees, 437 hoverflies and 1053 other flies, visiting apple blossoms. As expected, survey year and the number of apple flowers on transects were important variables explaining abundance and were retained in models for most groups of pollinators (Appendix S1: Tables S3–S8). Below we detail how habitat interventions and existing habitat variables affected each pollinator group. For solitary bees, wildflower habitat intervention was retained in the final model, with 26% greater numbers recorded in orchards with wildflower interventions (*z* = 2.35 *p* = 0.019) (Figure 1a) (Appendix S1: Table S3). There was an interaction between year and semi‐natural habitat to orchard ratio at 500 m (*z* = 3.26 *p* = 0.001) (Figure 1b). In the nesting intervention models, although the interaction between nesting intervention and proportion of bare‐ground was retained in all models it was not a significant factor (Appendix S1: Table S4).

**FIGURE 2:**
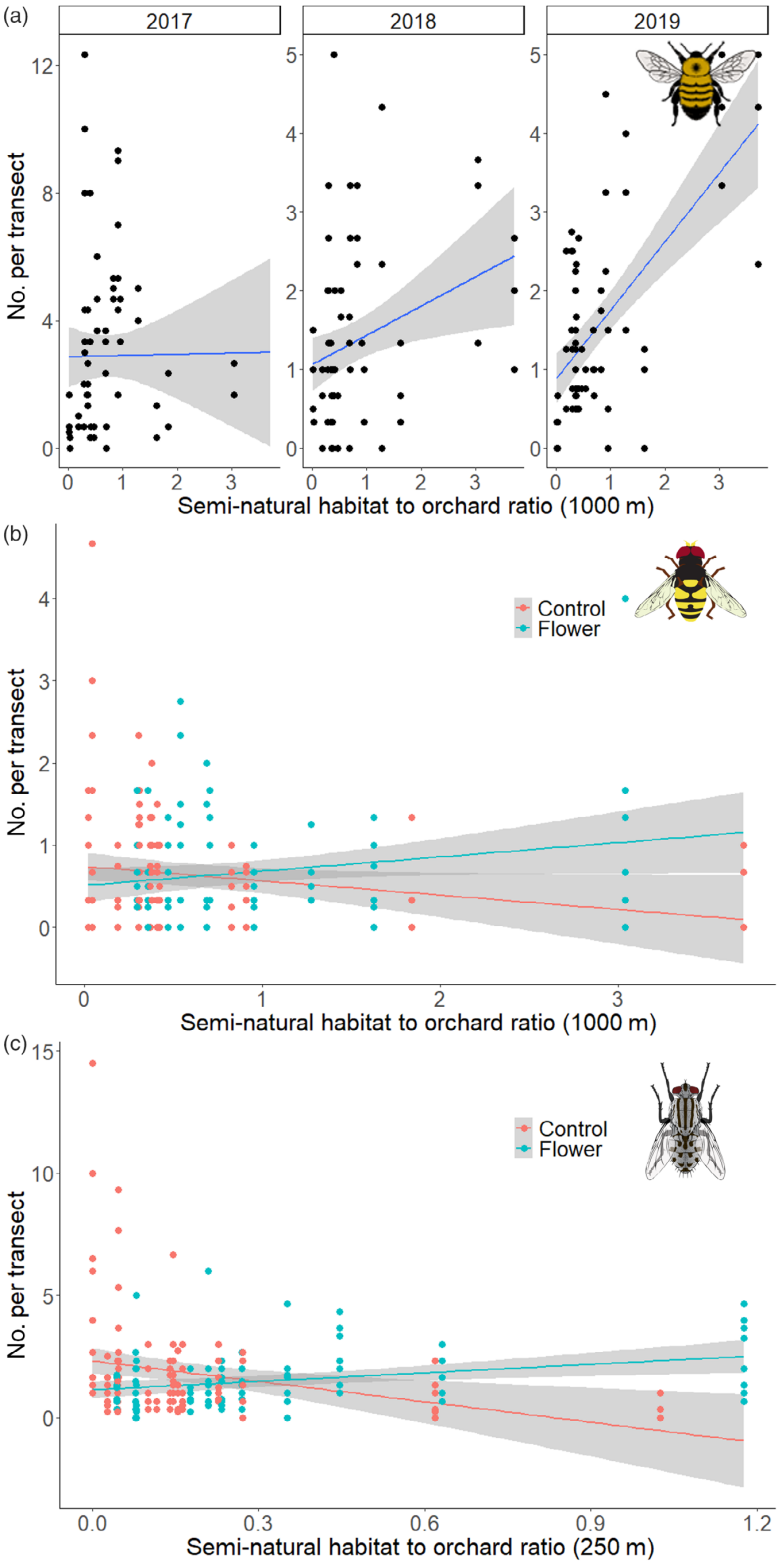
Relationship between abundance of pollinating insects visiting apple blossoms and (a) the semi‐natural habitat to orchard ratio within a 1000 m radius for bumblebees, (b) the semi‐natural habitat to orchard ratio within a 1000 m radius in orchards with (flower) and without (control) wildflower interventions for hoverflies, and (c) the semi‐natural habitat to orchard ratio within a 250 m radius in orchards with and without wildflower interventions for other flies (linear model with 95% confidence intervals shown).

For bumblebees, there was an interaction between year and semi‐natural habitat to orchard ratio at 1000 m (*z* = 2.46, *p* = 0.014) (Figure 2a) and an effect of proportion of key forage, which related to an increase in bumblebee abundance (*z* = 2.25, *p* = 0.024) (Appendix S1: Table S5). The abundance of hoverflies was significantly affected by the interaction between wildflower interventions and semi‐natural to orchard ratio at a 1000 m radius (Figure 2b) (Appendix S1: Table S6). Fly abundance decreased with an increased proportion of key forage (*z* = 2.55, *p* = 0.011), and an interaction between wildflower interventions and the semi‐natural to orchard ratio at 250 m was found (*z* = 3.50, *p* < 0.001) (Figure 2c) (Appendix S1: Table S7).

**FIGURE 3:**
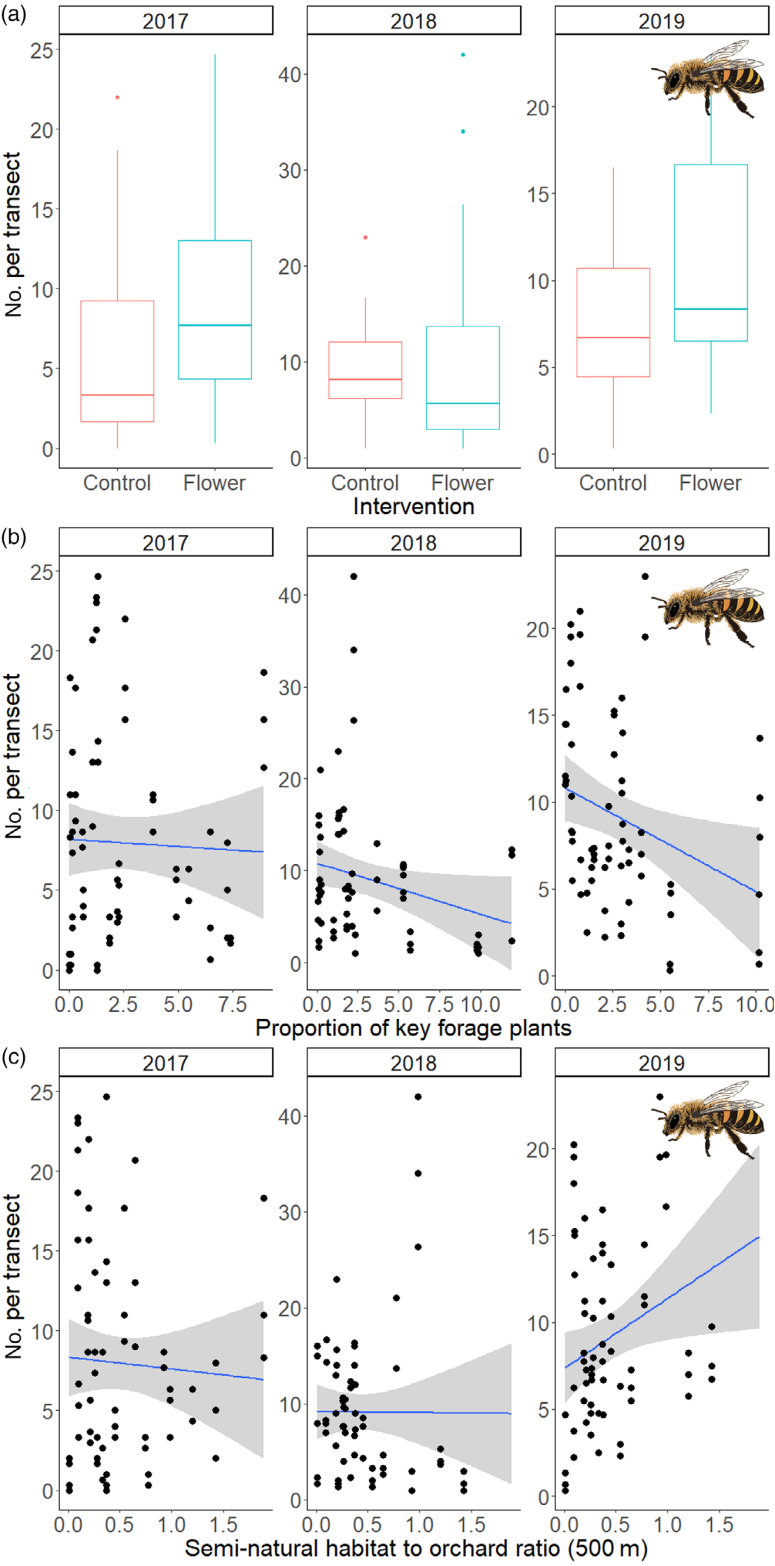
The relationship between honeybee abundance on apple blossoms and (a) wildflower habitat intervention (*n* = 11) versus control orchards (*n* = 12), (b) the proportion of key forage plants within the orchard boundary, and (c) the semi‐natural habitat to orchard ratio within a 500 m radius (linear model with 95% confidence intervals shown) across 23 apple orchards over 3 years.

For honeybee abundance, year interacted significantly with wildflower interventions (*z* = 3.14, *p* = 0.0017), proportion of key forage (*z* = 3.81, *p* < 0.001), and semi‐natural habitat to orchard ratios at 500 m (*z* = 2.90 *p* = 0.0038) (Appendix S1 Table S8), with the effects of these factors differing from year‐to‐year (Figure 3).

### Pollination deficits

**FIGURE 4:**
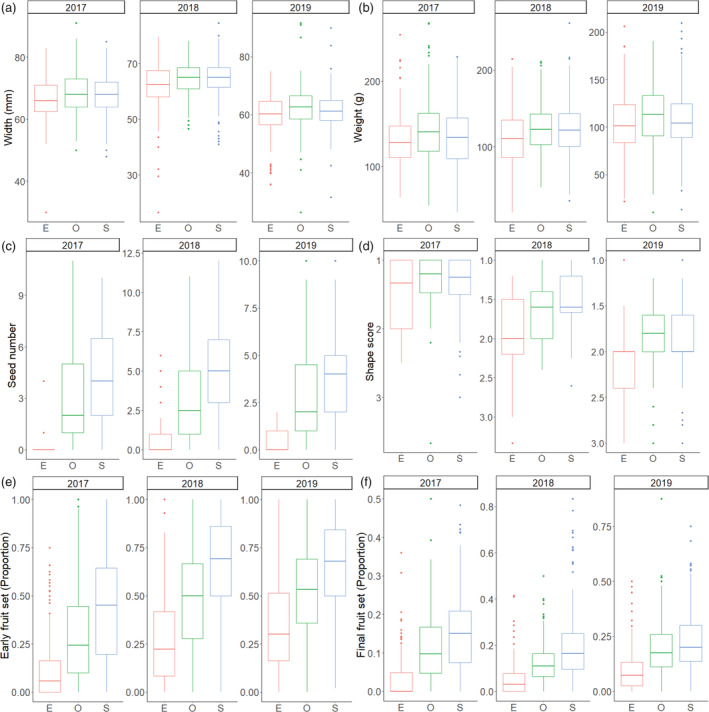
Effects of pollinator exclusion (E, pink bars), open pollination (O, green bars), and supplementary pollination (S, blue bars) on (a) apple size (maximum width cm), (b) weight (g), (c) seed number per apple, (d) mean shape score, (e) early fruit set, and (f) final fruit set across 23 apple orchards during the 3 years of the study.

Pollination treatment and year were significant in all models considering apple fruit set and quality metrics (Appendix S1: Tables S9 and S10). Open and supplementary pollination treatments increased apple size by 4.3% and 3.5% (*z* > 5.92, *p* < 0.001), weight by 9.8% and 6.8% (*z* > 4.32, *p* < 0.001), and seed number by 88.1% and 91.7% (*z* > 30.60, *p* < 0.001), improved average shape score by 14.5% and 17.6% (*z* > 5.23, *p* < 0.001), and early fruit set increased by 19.2% and 34.6% (*z* > 56.84, *p* < 0.001), and final fruit set by 8.7% and 13.3% (*z* > 36.58, *p* < 0.001), respectively, compared to when pollinators were excluded. The difference between supplementary pollination and open pollination was also significant for seed number (*z* = 14.52, *p* < 0.001) and initial (*z* = 42.55, *p* < 0.001) and final fruit set (*z* = 15.79, *p* < 0.001), indicating a deficit (Figure 4).

**FIGURE 5:**
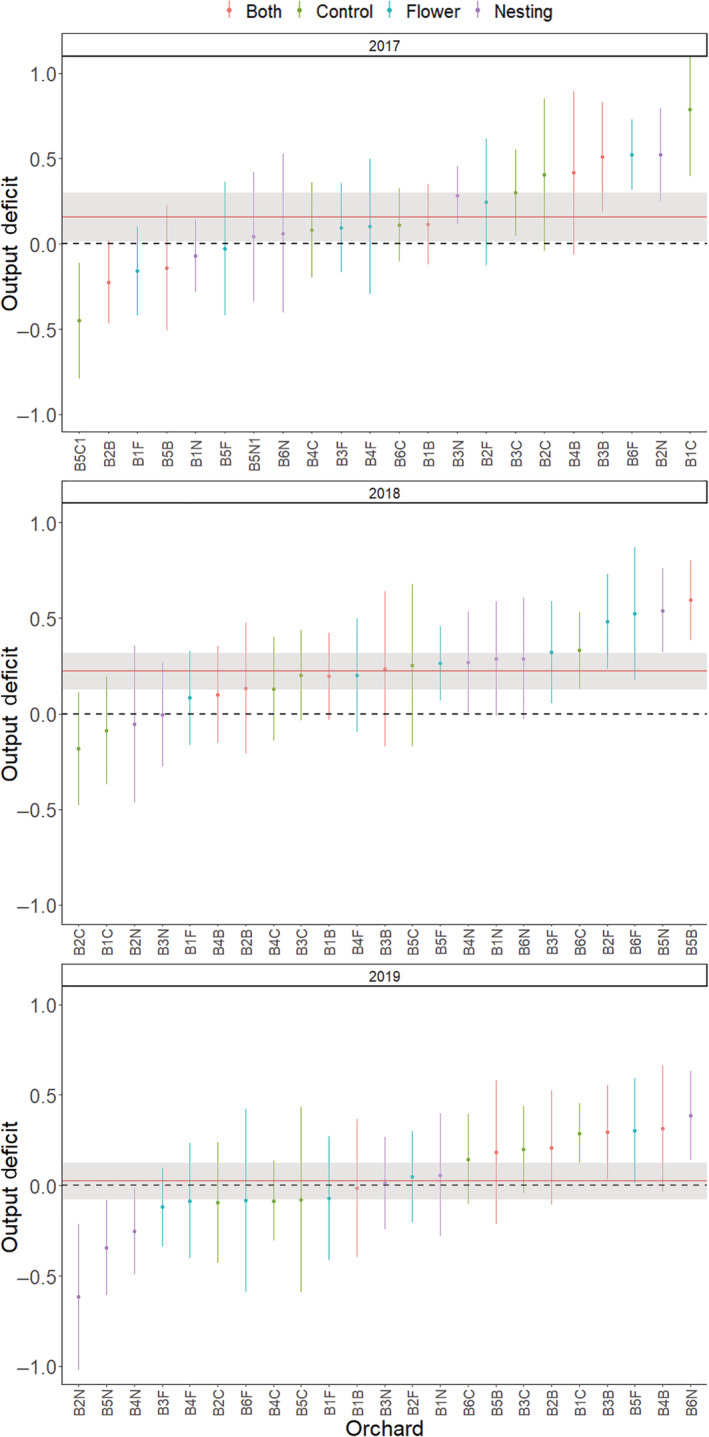
The extent of gross output deficits due to pollination ([supplementary pollination output − open pollination output]/maximum output) across 23 apple orchards studied over 3 years. Points show means and 95% CI for individual orchards colored by habitat intervention (flower = wildflower interventions, nesting = nesting interventions, both = a combination of wildflower and nesting interventions, control = no habitat intervention). Red horizontal lines and gray area show mean deficit and 95% CI for each year. The dashed lines show zero deficit where output under supplementary and open pollination treatments are equal.

When habitat intervention was considered (i.e., wildflower and nesting interventions), it was not found to be a significant factor in models considering gross output deficits, although there was an effect of year (*z* = 2.009, *p* = 0.044) (Appendix S1: Table S11). When flowering and nesting interventions were considered separately, again neither were retained in the final model although year remained significant (Nesting model: *z* = 1.985, *p* = 0.047) or close to significant (Flower model: *z* = 1.940, *p* = 0.052). Output deficits ranged from 22% (CI ± 8%) in 2018 to only 2.6% (CI ± 10%) in 2019. Within each year several individual orchards had output deficit estimates for which 95% CI did not overlap zero, indicating sub‐optimal pollination, with seven in 2017, of which one was negative (open pollination > supplementary pollination), seven in 2018 and eight in 2019, including three that were negative. Overall deficits across farms were greater than zero (95% CI) in 2017 and 2018, but not in 2019 (Figure 5).

### Orchard scale economic assessments

Overall insect pollinators contributed a mean increase in Gala apple net output of £15,887 (CI ± £1226) per hectare across all orchards in this study although this varied from year to year. A mean net economic deficit of £9132(CI ± £3873) was also recorded across all sites and years. The extent of this deficit varied between years and was smallest in 2019 at £1865 (CI ± £8458) and greatest in 2018 £14118 (CI ± £6582).

### Pollinators and pollination deficits

**FIGURE 6:**
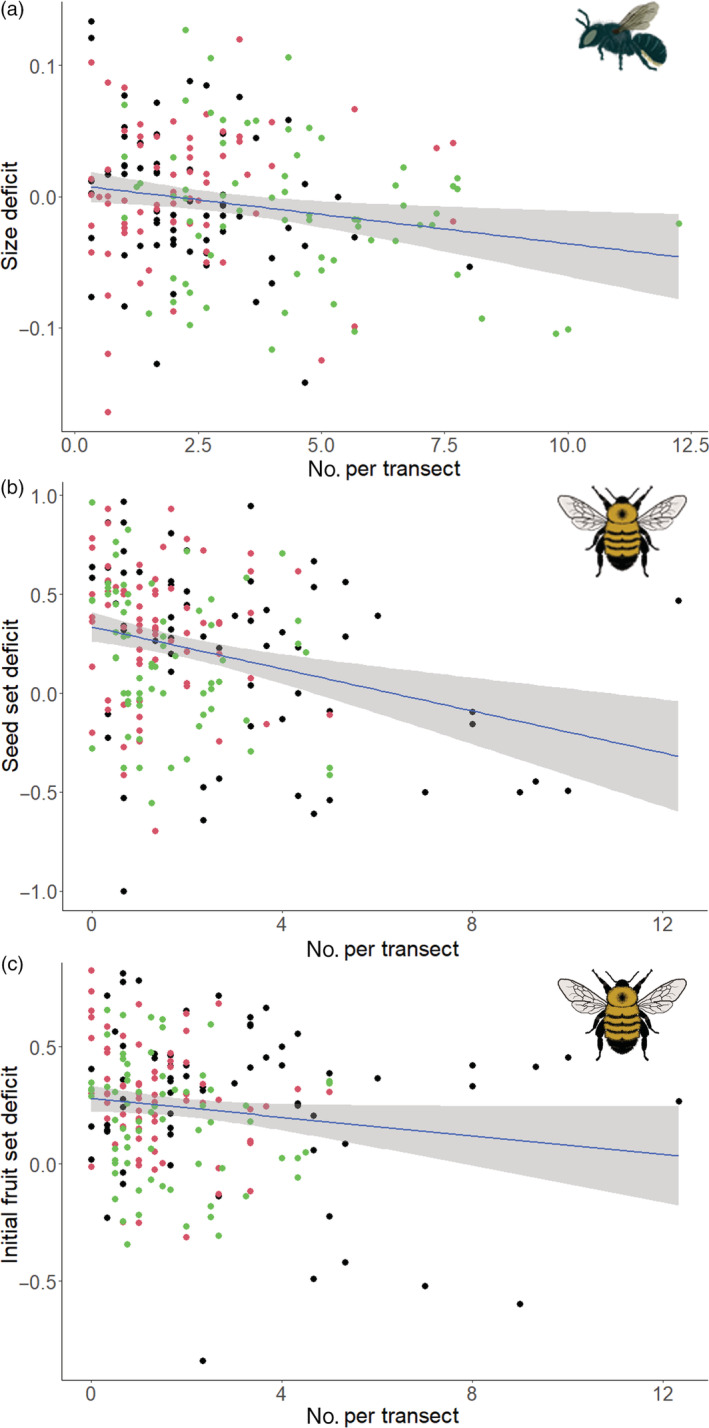
Relationship between (a) solitary bee abundance and fruit size deficits, (b) bumblebee abundance and seed set deficits, and (c) bumblebee abundance and initial fruit set deficits. Linear models with 95% CI shown. 2017 = black, 2018 = red, 2019 = green.

Year and abundance of hoverflies, flies, bumblebees, and honeybees on transects were retained in the model considering gross output deficits, although only year was significant (*z* = 2.72, *p* = 0.0064) (Appendix S1: Table S12). Solitary bees alone were retained in the final model for fruit size deficits, abundance of solitary bees was negatively related to fruit size deficit (*z* = 2.54, *p* = 0.011) (Appendix S1: Table S13) (Figure 6a). Bumblebees showed a negative (*z* = 3.28, *p* = 0.0011) relationship with seed set deficit, and several other factors were retained in the final model although none was significant (Appendix S1: Table S14) (Figure 6b). Year, bumblebees, and solitary bees were retained in the model for initial fruit set, with bumblebees significantly negatively associated with deficits (*z* = 2.34, *p* = 0.025) and greater fruit set deficits in 2017 compared to 2019 (Appendix S1: Table S15) (Figure 6c). Year, bumblebees, and hoverflies were retained in the model exploring final fruit set, although only year was significant with greater fruit set deficits again in 2017 compared to 2019 (Appendix S1: Table S16).

## DISCUSSION

Using an extensive network of experimentally manipulated UK commercial apple orchards, our 3‐year study demonstrates that deficits in apple outputs due to pollination exist. In some cases, these deficits amount to significant economic opportunities being missed, but their extent varies from year to year and from orchard to orchard. Increasing the abundance of wild pollinators, especially bumblebees and solitary bees, could reduce these deficits and deliver benefits for growers primarily through improvements in fruit quality including fruit size and seed set. Habitat interventions that provide non‐crop floral resources can increase the abundance of solitary bees observed visiting apple blossom. The abundance of pollinating insects including solitary bees, honeybees, bumblebees, hoverflies, and other flies was influenced by both local and landscape scale factors, which often varied from year to year. Bumblebees and hoverflies were influenced by the proportion of semi‐natural habitat at larger spatial scales (1000 m) while solitary bees, honeybees and other flies responded at 500 m or less. Wildflower interventions appeared to have a moderating effect on the abundance of hoverflies and other flies by countering the decline in abundance found in orchards located in areas with a high cover of semi‐natural habitat. We show that through targeted habitat creation and protection of local semi‐natural habitats, apple producers can increase abundance of key wild pollinators which could help meet their full production potential.

### Habitat interventions and apple pollinators

We recorded several insect pollinator groups visiting apple blossoms, including wild pollinators such as bumblebees, solitary bees, and flies alongside managed honeybees (Hutchinson et al., 2021; Pardo & Borges, 2020). Pollinator abundance on apple blossoms was influenced by several local and landscape‐scale variables depending on the pollinator group. Importantly, the wildflower habitat interventions we deployed for this study delivered increased numbers of solitary bees by an average of 26% on apple blossoms. The wildflower interventions were designed to provide pollen and nectar resources for wild bees outside the period of apple blossom (Heller et al., 2019) and as shown in a parallel study based on the same sites, they were visited by a diverse group of insects, including solitary bees from April through to August in each study year (Carvell et al., 2021). That the uplift in bee numbers did not interact with year indicates that benefits were delivered relatively quickly and served to increase bee numbers visiting apple blossom within one or two seasons, unlike the delayed benefits from habitat interventions observed in other studies (Albrecht et al., 2020; Blaauw & Isaacs, 2014), which are often considered a limitation preventing widespread adoption by growers.

We found no effects of nesting interventions or availability of existing bare‐ground on solitary bees, indicating that availability of such a habitat is not currently a constraint on bee populations. However, this may not be the case if other resources such as forage become widely available, in which case increasing nesting habitats may deliver benefits. The use of herbicides, which can have additional negative effects, may not be necessary to create sites for ground nesting bees, and alternative means such as mechanical excavation (Gregory & Wright, 2005) or depositing appropriate soil (Fortel et al., 2016) have potential. Although this would need to be tested within an orchard context. Solitary bee numbers were influenced by the relative availability of semi‐natural habitats at intermediate scales (500 m), and this effect varied from year to year but was positive in 2018 and 2019, likely due to increased availability of nesting and forage sites (Földesi et al., 2016; Joshi et al., 2016). Importantly there was no evidence of competition for floral resources and locally available spring‐flowering plants did not reduce the number of solitary bees visiting apple blossoms (Nicholson et al., 2019).

Bumblebee abundance on apple blossoms was positively related to the proportion of semi‐natural habitats at the largest spatial scale investigated, 1000 m. Larger bees such as bumblebees typically forage over greater distances than smaller species (Greenleaf et al., 2007). Semi‐natural areas, including woodland and low input grassland, are preferred nesting sites for many bumblebee species from which they can visit apple blossom during the spring. The predominance of landscape factors determining wild bee abundance in apples has been observed in another study (Bartholomée et al., 2020). We found that the abundance of flies, including hoverflies, on apple flowers was also influenced by the proportion of semi‐natural habitat. Although their response was moderated by the presence of the wildflower interventions which counteracted the negative effects of increasing semi‐natural habitat on abundance. This suggests that alternative and potentially more attractive floral resources provided by non‐cropped habitats at intermediate scales may draw flies away from apple blossom (Nicholson et al., 2019), but the presence of wildflower interventions near to the orchards appeared to mitigate this effect on flies, perhaps due to local spill over from the flower plots into the orchards (Albrecht et al., 2020).

The response of honeybees to wildflower habitat interventions and local and landscape variables was complex and varied from year to year. We observed both increases and decreases in activity in response to our wildflower interventions depending on year. Activity on apple blossoms was also reduced in the presence of a high proportion of local floral resources in and around the orchard. Honeybees use recruitment and will visit flowers *en masse*. Other research has shown that when alternative resources are available they can be drawn away from flowers, including apples (Osterman et al., 2021). While some growers involved in our study host honeybees on their farms, they are not used in every orchard. Our study shows that honeybees are common visitors to apples blossom but visitation is unpredictable, and factors which determine their abundance vary from season to season.

### Extent of pollination deficits

We confirm the critical role of pollinators for both apple yield and quality (Garratt et al., 2014; Samnegård, Hambäck, & Smith, 2019) with the exclusion of pollinators reducing fruit set, weight and quality parameters, equivalent to an average of more than £15 k net output per hectare, although the extent of this benefit varied between years. Although we identify deficits where fruit set and quality are lower in open compared to supplementary pollination (initial fruit set, final fruit set and seed number), this did not mean yield and resulting economic deficits were ubiquitous, and the extent of deficits varied across orchards, and from year to year. For example, the mean deficit was 22% in 2018 compared to only 2.6% in 2019. The extent of a pollination deficit will be determined by the availability of pollinators but also how this interacts with grower practices, particularly fruit thinning. Thinning is carried out to avoid too many fruit on trees to maximize output by balancing fruit number and fruit quality (Link, 2000). Those orchards in our study that present a negative pollination deficit are at maximum fruit load where additional pollination could reduce yield and quality through resource limitations in the trees resulting in smaller fruit (Garratt et al., 2021; Link, 2000). Nonetheless, on average, deficits are positive across our orchards, and significantly above zero in 2017 and 2018, indicating most are at risk of under rather than over pollination. That our habitat interventions had no significant effects on the extent of these deficits may be because the uplift in solitary bee numbers resulting from wildflower interventions was not sufficient to increase pollination and reduce deficits measurably. Perhaps the size of the wildflower planting in our landscape context was not sufficient (Faichnie et al., 2021), although larger flower plots in some contexts have been found to act as a pollinator sink, reducing visitors to crop flowers (Krimmer et al., 2019). Alternatively, the lack of an effect could be because substantial pollination is being delivered by other pollinators that did not respond to our habitat interventions (e.g., bumblebees). Finally, previous research found that benefits of floral plantings on fruit production were only seen in year 3 and 4 (Blaauw & Isaacs, 2014), and flower margins that have been established for a longer time improve pollination (Albrecht et al., 2020). In our study, positive effects on solitary bees were observed from the first year after sowing, although these were maybe insufficient to increase pollinator numbers to a level where benefits to production were detected.

### Pollinators and pollination deficits

Deficits in total gross output and individual metrics of fruit quality were apparent, but groups of pollinators have the potential to reduce shortfalls in particular metrics and help growers achieve their output potential. Increased bumblebee numbers reduced deficits in initial fruit set and seed number. Sufficient initial fruit set is important to allow for adequate fruit to develop to achieve a good yield, although the tree will shed much of this fruit and a portion will be removed through thinning (Link, 2000). Greater initial fruit set may be important in years when fruit numbers are reduced through late season frost events (Unterberger et al., 2018) or in years where fruit set is particularly low. Good seed set results in apples of improved quality, particularly size and shape (Brookfield et al., 1996; Garratt et al., 2021; Matsumoto et al., 2012) and increased bumblebee abundance was associated with reduced deficits in seed number per apple. Bumblebees forage over large distances (Greenleaf et al., 2007; Redhead et al., 2016) and foraging could bring them in to contact with compatible pollen beyond polliniser trees planted within the orchards, including wild crab apples and compatible cultivars in neighboring orchards (Matsumoto, 2014), therefore increasing their contribution to pollination and seed set. We observed that deficits in apple size, a key metric of fruit quality, were reduced at sites with a greater abundance of solitary bees. Apple width is used to assign apples to classes for which growers can get an improved price (Garratt et al., 2014). The foraging behavior and improved efficiency of solitary bees compared to other pollinators in apples (Russo et al., 2017) may be improving pollination or the distribution of fruit on trees, thus maximizing size.

Managed honeybees are employed as pollinators of apples in many parts of the world. Interestingly, we found no relationship between honeybees and the extent of deficits in this study. Honeybees are sometimes less effective than other pollinators, visiting flowers primarily for nectar and side‐working (Russo et al., 2017), but honeybees can improve pollination in apples (Geslin et al., 2017; Rollin & Garibaldi, 2019) in certain contexts. However, the transient nature of their foraging seen in this study may reduce their capacity to deliver consistent improvements in pollination in our orchards.

### Prospects for management of pollination in orchards

Our study demonstrates the important role of pollinators in underpinning economic output in apple orchards, contributing on average over £16 k per hectare. The important role of wild bees, including solitary bees and bumblebees, for pollinating UK apples is highlighted. By improving fruit set and fruit quality, pollinators can help growers achieve their maximum potential. There was no direct effect of habitat interventions (nesting or floral) on the extent of deficits, but the introduction of wildflower habitats adjacent to orchards increased numbers of solitary bees visiting apple flowers during bloom and could deliver benefits in years where pollination is particularly poor.

The ratio of semi‐natural habitats, including woodland and low‐input grasslands, to orchards within 500 and 1000 m was also positively associated with increased solitary bee and bumblebee numbers, respectively, in 2 years of this study. The importance of proximity (within 500 m) to non‐cropped habitats for solitary bees in apples has previously been shown (Földesi et al., 2016; Joshi et al., 2016). That no interactive effect on wild bees between habitat interventions and landscape context was found indicates that uplifts in bee numbers due to both factors will likely be additive. Given the average orchard size was ~2 ha and landscape context at a 500 m or greater radius was most influential, overall orchard size could remain relatively unchanged but the need for uncropped semi‐natural habitats within the local landscape is evident. Locally available co‐flowering resources are sometimes considered a risk to pollination as they may compete with apple flowers for pollinators (Nicholson et al., 2019; Osterman et al., 2021). Only flies and honeybees, and only in some years, were affected by the availability of local co‐flowering resources in our study. Therefore, we consider the risk of competition on apple pollination to be low. Floral resources that support pollinators within orchards should be encouraged due to the overall benefits they may provide, including to pest control for example (Campbell et al., 2017). Through targeted habitat creation and protection of local semi‐natural habitats, apple producers can increase abundance of key wild pollinators which could help meet their full production potential.

## AUTHOR CONTRIBUTIONS

Michael P. D. Garratt, Rory O'Connor, Claire Carvell, Michelle T. Fountain, Tom D. Breeze, Richard Pywell, Mike Edwards, Marek Nowakowski and Simon G. Potts designed the study, Rory O'Connor, Claire Carvell, Michelle T. Fountain, Tom D. Breeze, John Redhead, Lois Kinneen, Nadine Mitschunas, Louise Truslove, Celina Xavier e Silva, Claire Brittain and Michael P. D. Garratt collected and curated data, Michael P. D. Garratt, Tom D. Breeze, John Redhead and Lois Kinneen, analyzed the data, Michael P. D. Garratt, Claire Carvell, Michelle T. Fountain, Tom D. Breeze, Richard Pywell, Nigel Jenner, Caroline Ashdown, Claire Brittain, Megan McKerchar, Charnee Butcher and Peter Sutton secured funding; Michael P. D. Garratt, Claire Carvell, Michelle T. Fountain, Tom D. Breeze, Richard Pywell, John Redhead and Simon G. Potts wrote the original draft; Rory O'Connor, Lois Kinneen, Nadine Mitschunas, Louise Truslove, Celina Xavier e Silva, Nigel Jenner, Caroline Ashdown, Claire Brittain, Megan McKerchar, Charnee Butcher, Mike Edwards, Marek Nowakowski and Peter Sutton reviewed and edited drafts. All authors gave final approval for publication and agree to be held accountable for the work performed therein.

## CONFLICT OF INTEREST

The authors declare no conflict of interest.

## Supporting information


Appendix S1

## Data Availability

Data (Garratt et al., 2022) are archived in the University of Reading data archive at https://doi.org/10.17864/1947.000400.
