# Supplementary material for: Addressing pollination deficits in orchard crops through habitat management for wild pollinators
Source: Ecol Appl. 2022 Nov 30;33(1):e2743. doi: 10.1002/eap.2743 (PMC10078601; doi:10.1002/eap.2743)
Supplement: Supplementary file 1 — Appendix S1 [file EAP-33-0-s001.pdf]

Ecological Applications

Appendix S1

Addressing pollination deficits in orchard crops through habitat management for wild pollinators

Michael P. D. Garratt, Rory O'Connor, Claire Carvell, Michelle T. Fountain, Tom D. Breeze, Richard Pywell, John Redhead, Lois Kinneen, Nadine Mitschunas, Louise Truslove, Celina Xavier e Silva, Nigel Jenner, Caroline Ashdown, Claire Brittain, Megan McKerchar, Charnee Butcher, Mike Edwards, Marek Nowakowski, Peter Sutton, and Simon G. Potts

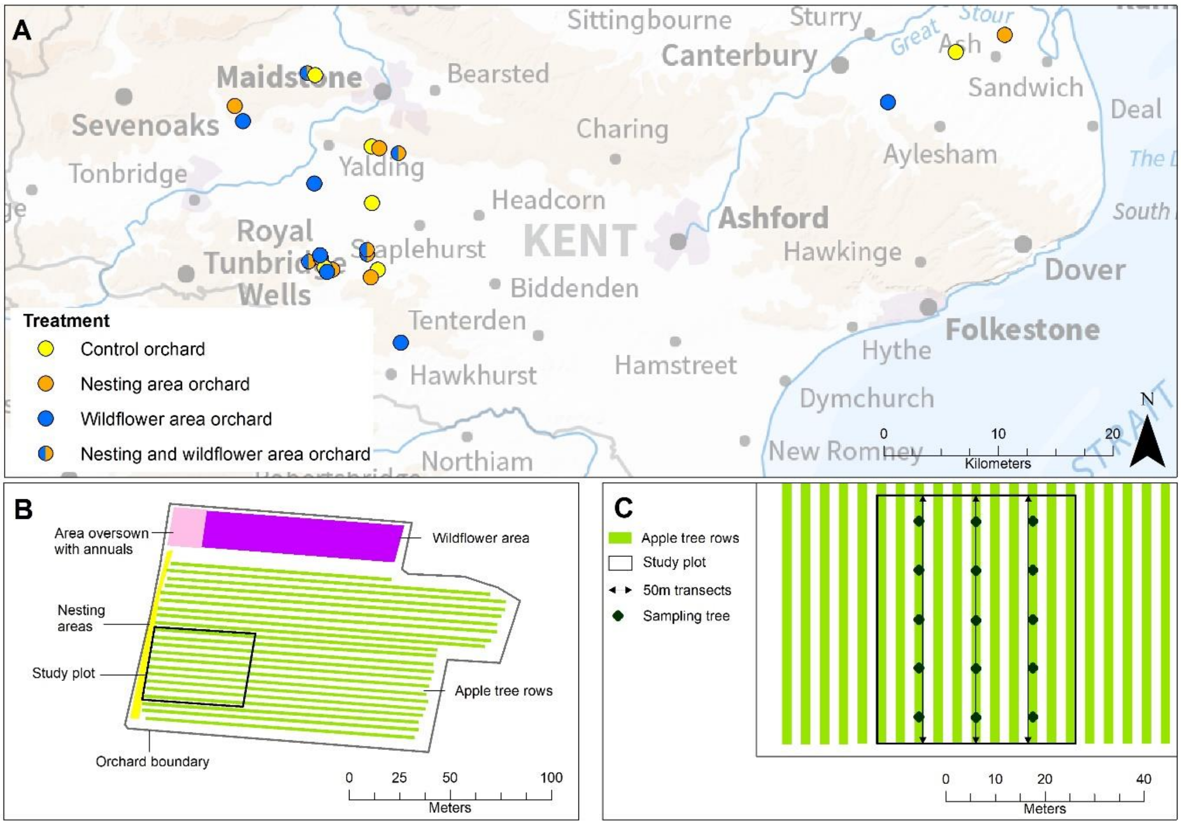

Fig S1. A. Study orchard locations in Kent in south-eastern England and their associated habitat intervention treatments. B. Example study orchard showing flower plots with annual and perennial sowing and extended herbicide sprayed nesting area. C. Example study plot located in each orchard from which pollinator surveys and pollination service assessments were made.

Table S1. Flower species and the percentage of the mix in the wildflower intervention areas including perennial, and annual species sown in 2016/2017.

Vegetation component	Species	% of mix
Perennial mix	*Achillea millefolium* (Yarrow)	0.83
	Anthyllis vulneraria (Kidney Vetch)	0.83
	Barbarea vulgaris (Winter-cress)	3.33
	Centaurea nigra (Common Knapweed)	5.00
	Daucus carota (Wild Carrot)	1.67
	Leontodon hispidus (Rough Hawkbit)	3.33
	Leucanthemum vulgare (Oxeye Daisy)	1.67
	Lotus corniculatus (Birdsfoot Trefoil)	3.33
	Plantago media (Hoary Plantain)	0.83
	Primula veris (Cowslip)	1.67
	Ranunculus acris (Meadow Buttercup)	5.00
	Reseda lutea (Wild Mignonette)	0.83
	Silene dioica (Red Campion)	0.83
	Taraxacum officinale (Dandelion)	3.33
	Trifolium pratense (Red Clover)	0.83
	Sown perennial flowers TOTAL (4 kg/ha)	33.33
	Basic fine grass mix (8 kg/ha)	67.00
Annual mix [b]	*Alliaria petiolata* (Garlic Mustard)	10.00
	Anthemis austriaca (Corn Chamomile)	5.00
	Brassica spp. (Winter oilseed rape)	10.00
	Camelina sativa (Gold of Pleasure)	15.00
	Centaurea cyanus (Cornflower)	10.00
	Echium vulgare (Viper's Bugloss)	10.00
	Glebionis segetum (Corn Marigold)	5.00
	Papaver rhoeas (Common Poppy)	10.00
	Raphanus sativus (Fodder raddish)	10.00
	Trifolium incarnatum (Crimson Clover)	15.00
	Sown annual flowers TOTAL (10 kg/ha)	100

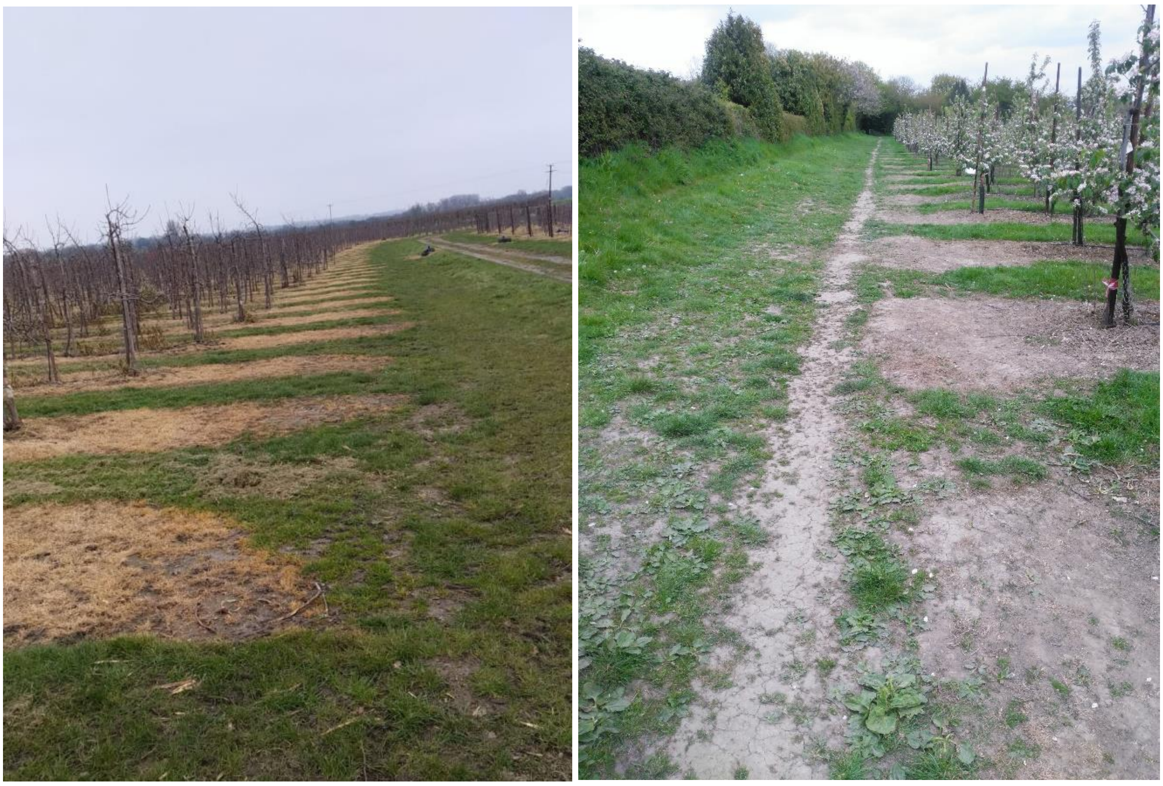

Fig S2. Nesting interventions created in the study orchards which involved extending the herbicide treated area at the end of each tree row by between 2.2 and 3.3 m^2.

Testing for spatial autocorrelation

The effect of landscape context on pollinators was explored at multiple scales (250, 500 and 1000 m from the orchard centre) which were often larger than the separation between individual orchards, we tested the pollinator abundance data for spatial autocorrelation. Abundance was averaged across transects and across years for each orchard and a Moran's I test was carried out. No significant autocorrelation was found for solitary bees, honeybees, bumblebees, or hoverflies whilst flies showed significant autocorrelation (SI Table 2). Inspection of the data showed that the three orchards with the lowest abundance of flies were clustered and located away from most other orchards (block 6, SI Fig 3). When the test was re-run with blocks 1 to 5 only, no significant autocorrelation was found for flies. For the analysis we included block as a random factor in all models, which accounts for the autocorrelation seen for flies driven by block 6.

Table S2. Moran's I and associated p values for the abundance of different pollinator groups recorded in study orchards

Guild	Stat	MI	P value	Centile
Bumblebee	Morans.InvD	-0.0036	0.5795	0.772
Other fly	Morans.InvD	0.3067	1.60E-06	0.998
Honeybee	Morans.InvD	0.0379	0.3327	0.868
Hoverfly	Morans.InvD	-0.0726	0.7084	0.360
Solitary bee	Morans.InvD	0.0957	0.0957	0.948

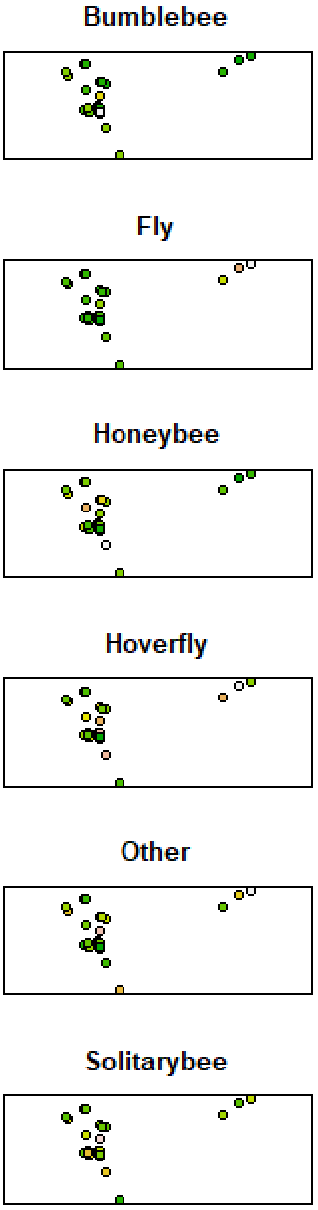

Fig S3. Points showing location and relative abundance of flower visitors by group recorded during the study. Green represents high relative abundance through to white showing low abundance.

Table S3. Linear mixed model average model output for solitary bees in response to wildflower habitat interventions and existing floral and nesting resources at local and landscape scales. Models including the semi-natural habitat to orchard ratio at a 500m radius had the lowest AIC value and are shown here. All models within <2AIC of the global model averaged and full model-averaged coefficients shown.

Variable	Estimate	SE	z value	Pr(>|z|) d
WildflowerIntervention	0.27253	0.11545	2.351	0.0187 *
Year2018	-0.11312	0.06493	1.731	0.0833.
Year2019	0.48756	0.06251	7.751	< 0.001 ***
FlowersPerTransect	0.24968	0.06349	3.908	< 0.001 ***
SemiNaturalOrchardRatio500	-0.28540	0.09644	2.941	0.00323 **
Year2018:SemiNaturalOrchardRatio500	0.24538	0.13398	1.820	0.0687.
Year2019:SemiNaturalOrchardRatio500	0.44800	0.13686	3.255	0.00114 **
WildflowerIntervention:Year2018	-0.08640	0.12979	0.664	0.506
WildflowerIntervention:Year2019	-0.11415	0.15492	0.735	0.462
ProportionKeyForage	0.02350	0.04805	0.487	0.626

Controls with no habitat interventions and 2017 (Year) are used as baseline levels for the models
Positive estimates indicate higher numbers e.g. higher insect counts in wildflower intervention orchards than control orchards
Significance levels: . < 0.01, * <0.05, ** <0.01, ***<0.001.

Table S4. Linear mixed model average model output for solitary bees in response to nesting habitat interventions and existing floral and nesting resources at local and landscape scales. Models including the semi-natural habitat to orchard ratio at a 500m radius had the lowest AIC value and are shown here. All models within <2AIC of the global model averaged and full model-averaged coefficients shown.

Variable	Estimate	SE	z value	Pr(>|z|) d
Year2018	-0.12285	0.06635	1.840	0.065737 .
Year2019	0.48756	0.06251	7.751	< 2e-16 ***
FlowersPerTransect	0.22853	0.06344	3.580	0.000343 ***
ProportionBareground	0.09360	0.07748	1.204	0.228769
SemiNaturalOrchardRatio500	-0.23251	0.10695	2.161	0.030725 *
SemiNaturalOrchardRatio500	-0.23251	0.10695	2.161	0.030725 *
Year2018:SemiNaturalOrchardRatio500	0.22055	0.13812	1.587	0.112442
Year2019:SemiNaturalOrchardRatio500	0.46621	0.12985	3.568	0.000359 ***
NestingIntervention	0.02834	0.06806	0.414	0.678519
NestingIntervention:ProportionBareground	-0.02283	0.07735	0.294	0.768420
ProportionBareground:SemiNaturalOrchardRatio500	0.01174	0.06202	0.188	0.850549
NestingIntervention:SemiNaturalOrchardRatio500	-0.03068	0.10362	0.295	0.767627

Controls with no habitat interventions and 2017 (Year) are used as baseline levels for the models
Significance levels: . < 0.01, * <0.05, ** <0.01, ***<0.001.

Table S5. Linear mixed model average model output for bumblebees in response to habitat interventions and existing floral and nesting resources at local and landscape scales. Models including the semi-natural habitat to orchard ratio at a 1000m radius had the lowest AIC value and are shown here. All models within <2AIC of the global model averaged and full model-averaged coefficients shown.

Variable	Estimate	SE	z value	Pr(>|z|) d
WildflowerIntervention	-0.04168	0.12209	0.339	0.7343
Year2018	-0.46372	0.06914	6.666	< 2e-16 ***
Year2019	-0.27527	0.06637	4.122	3.75e-05 ***
FlowersPerTransect	0.33532	0.06996	4.764	1.90e-06 ***
ProportionKeyForage	0.20127	0.08886	2.254	0.0242 *
SemiNaturalOrchardRatio1000	0.06770	0.17259	0.390	0.6966
WildflowerIntervention:ProportionKeyForage	0.29652	0.21870	1.352	0.1763
Year2018:SemiNaturalOrchardRatio1000	0.32685	0.16768	1.938	0.0526 .
Year2019:SemiNaturalOrchardRatio1000	0.40077	0.16207	2.458	0.0140 *
ProportionKeyForage:SemiNaturalOrchardRatio1000	0.03483	0.14235	0.244	0.8076

Controls with no habitat interventions and 2017 (Year) are used as baseline levels for the models
Significance levels: . < 0.01, * <0.05, ** <0.01, ***<0.001.

Table S6. Linear mixed model average model output for hoverflies in response to habitat interventions and existing floral resources at local and landscape scales. Models including the semi-natural habitat to orchard ratio at a 1000m radius had the lowest AIC value and are shown here. All
models within <2AIC of the global model averaged and full model-averaged coefficients shown.

Variable	Estimate	SE	z value	Pr(>|z|) d
WildflowerIntervention	0.086432	0.071621	1.200	0.230299
Year2018	0.192551	0.054850	3.489	0.000485 ***
Year2019	0.241313	0.056270	4.263	2.02e-05 ***
ProportionKeyForage	-0.060166	0.094881	0.632	0.527658
SemiNaturalOrchardRatio1000	-0.087812	0.107065	0.815	0.415044
WildflowerIntervention:SemiNaturalOrchardRatio1000	0.378881	0.136974	2.749	0.005971 **
Year2018:SemiNaturalOrchardRatio1000	0.140357	0.149666	0.933	0.350916
Year2019:SemiNaturalOrchardRatio1000	-0.131209	0.151038	0.864	0.387447
ProportionKeyForage:SemiNaturalOrchardRatio1000	-0.383116	0.246467	1.549	0.121428
Year2018:ProportionKeyForage	0.068715	0.116706	0.587	0.556902
Year2019:ProportionKeyForage	0.095529	0.152895	0.624	0.532791
FlowersPerTransect	0.006877	0.025935	0.264	0.791681
WildflowerIntervention:Year2018	0.004710	0.043146	0.109	0.913575
WildflowerIntervention:Year2019	-0.019200	0.063143	0.303	0.761722

Controls with no habitat interventions and 2017 (Year) are used as baseline levels for the models
Significance levels: . < 0.01, * <0.05, ** <0.01, ***<0.001.

Table S7. Linear mixed model average model output for flies in response to habitat interventions and existing floral resources at local and landscape scales. Models including the semi-natural habitat to orchard ratio at a 250m radius had the lowest AIC value and are shown here. All models within <2AIC of the global model averaged and full model-averaged coefficients shown.

Variable	Estimate	SE	z value	Pr(>|z|) d
WildflowerIntervention	-0.033997	0.066489	0.508	0.611171
Year2018	0.452806	0.062461	7.205	< 2e-16 ***
Year2019	0.080774	0.062347	1.288	0.197897
ProportionKeyForage	-0.155913	0.060811	2.549	0.010804 *
SemiNaturalOrchardRatio250	-0.025857	0.061373	0.419	0.675404
WildflowerIntervention:SemiNaturalOrchardRatio250	0.450570	0.128570	3.504	0.000458 ***
WildflowerIntervention:Year2018	0.033531	0.086473	0.387	0.698958
WildflowerIntervention:Year2019	-0.009717	0.059704	0.162	0.871425
WildflowerIntervention:ProportionKeyForage	0.020870	0.072793	0.286	0.775236
ProportionKeyForage:SemiNaturalOrchardRatio250	-0.018676	0.081822	0.227	0.820274

Controls with no habitat interventions and 2017 (Year) are used as baseline levels for the models
Significance levels: . < 0.01, * <0.05, ** <0.01, ***<0.001.

Table S8. Linear mixed model average model output for honeybees in response to habitat interventions and existing floral resources at local and landscape scales. Models including the semi-natural habitat to orchard ratio at a 500m radius had the lowest AIC value and are shown here. All models within <2AIC of the global model averaged and full model-averaged coefficients shown.

Variable	Estimate	SE	z value	Pr(>|z|) d
WildflowerIntervention	0.64491	0.21450	2.988	0.002804**
Year2018	0.16945	0.09920	1.697	0.089656.
Year2019	0.28427	0.09452	2.988	0.002804**
FlowersPerTransect	0.39608	0.10610	3.709	0.000208***
ProportionKeyForage	0.35442	0.19712	1.787	0.073870.
SemiNaturalOrchardRatio500	-0.14641	0.20692	0.703	0.481799
WildflowerIntervention:Year2018	-0.78792	0.24937	3.143	0.001670**
WildflowerIntervention:Year2019	-0.52436	0.21762	2.395	0.016642*
WildflowerIntervention:ProportionKeyForage	-0.49003	0.35102	1.392	0.163944
Year2018:ProportionKeyForage	-0.87045	0.22700	3.811	0.000138***

Year2019:ProportionKeyForage	-0.57972	0.26011	2.215	0.026783*
Year2018:SemiNaturalOrchardRatio500	0.01000	0.26026	0.038	0.969503
Year2019:SemiNaturalOrchardRatio500	0.67853	0.23306	2.895	0.003792**
ProportionKeyForage:SemiNaturalOrchardRatio500	-0.28014	0.27865	1.003	0.315986
WildflowerIntervention:SemiNaturalOrchardRatio500	-0.23611	0.35662	0.660	0.509213

Controls with no habitat interventions and 2017 (Year) are used as baseline levels for the models
Significance levels: . < 0.01, * <0.05, ** <0.01, ***<0.001.

Table S9. Linear mixed model output for fruit quality parameters in response pollination treatments and study year. A single model resulted from the model selection procedure, outputs from which are presented here.

Variable	Treatment level	Estimate	SE	t value	Pr(>|z|) d
Width	Intercept	66.1626	0.5383	122.903	< 2e-16 ***
	Open	2.2507	0.3143	7.162	1.08e-12 ***
	Supplementary	1.8999	0.3211	5.917	3.78e-09 ***
	Year2018	-3.6013	0.3181	-11.320	<2e-16 ***
	Year2019	-6.0740	0.3179	-19.107	< 2e-16 ***
Weight	Intercept	130.599	2.722	47.983	5.35e-15 ***
	Open	10.064	1.585	6.348	2.64e-10 ***
	Supplementary	6.990	1.620	4.316	1.66e-05 ***
	Year2018	-15.340	1.607	-9.543	<2e-16 ***
	Year2019	-26.670	1.606	-16.602	< 2e-16 ***
Shape	Intercept	1.54434	0.04103	37.642	< 2e-16 ***
	Open	-0.20409	0.03903	-5.229	2.47e-07 ***
	Supplementary	-0.25094	0.03907	-6.423	3.01e-10 ***
	Year2018	0.29601	0.03994	7.412	4.72e-13 ***
	Year2019	0.57754	0.03979	14.513	< 2e-16 ***

Pollinator exclusion treatment and 2017 (Year) are used as baseline levels for the models
Significance levels: . < 0.01, * <0.05, ** <0.01, ***<0.001.

Table S10. Generalised linear mixed model output for seed number and fruit set parameters in response pollination treatments and study year. A single model resulted from the model selection procedure, outputs from which are presented here.

Variable	Treatment level	Estimate	SE	z value	Pr(>|z|) d
Seed number	Intercept	-1.10219	0.09964	-11.062	< 2e-16 ***
	Open	2.15427	0.07041	30.598	< 2e-16 ***
	Supplementary	2.50862	0.06989	35.895	< 2e-16 ***
	Year2018	0.09745	0.03011	3.236	0.00121 **
	Year2019	-0.11441	0.03165	-3.615	0.00030 ***
Early fruit set	Intercept	-2.03749	0.19964	-10.21	<2e-16 ***
	Open	0.96094	0.01690	56.84	<2e-16 ***
	Supplementary	1.64433	0.01790	91.86	<2e-16 ***
	Year2018	0.90730	0.01749	51.87	<2e-16 ***
	Year2019	1.06698	0.01796	59.41	<2e-16 ***
Final fruit set	Intercept	-3.25896	0.09649	-33.774	< 2e-16 ***
	Open	1.03575	0.02831	36.580	< 2e-16 ***
	Supplementary	1.37094	0.02832	48.404	< 2e-16 ***
	Year2018	0.11360	0.02613	4.348	1.38e-05 ***
	Year2019	0.53647	0.02538	21.138	< 2e-16 ***

Pollinator exclusion treatment and 2017 (Year) are used as baseline levels for the models
Significance levels: . < 0.01, * <0.05, ** <0.01, ***<0.001.

Table S11. Linear mixed model average model output for gross output deficits in response to habitat interventions and existing floral and nesting resources at local and landscape scales. Models including the semi-natural habitat to orchard ratio at a 1000m radius had the lowest AIC value and are shown here. All models within <2AIC of the global model averaged and full model-averaged coefficients shown.

Variable	Estimate	SE	z value	Pr(>|z|) d
Year2018	0.103937	0.058220	1.774	0.07605
Year2019	-0.121470	0.060090	2.009	0.04449
SemiNaturalOrchardRatio1000	-0.107018	0.142791	0.748	0.45426
Year2018:SemiNaturalOrchardRatio1000	0.128799	0.171255	0.751	0.45267
Year2019:SemiNaturalOrchardRatio1000	0.115224	0.157596	0.730	0.46548
ProportionBareground	0.006876	0.031720	0.216	0.82936
ProportionKeyForage	-0.019602	0.040236	0.485	0.62767
ProportionBareground:ProportionKeyForage	0.057490	0.109133	0.526	0.59882

Controls with no habitat interventions and 2017 (Year) are used as baseline levels for the models
Significance levels: . < 0.01, * <0.05, ** <0.01, ***<0.001.

Table S12. Linear mixed model average model output for gross output deficits in response to abundance of different pollinator groups. All models within <2AIC of the global model averaged and full model-averaged coefficients shown.

Variable	Estimate	SE	z value	Pr(>|z|) d
Year2018	0.026099	0.069745	0.372	0.709702
Year2019	-0.186042	0.067964	2.723	0.006478 **
Hoverfly	0.332028	0.170641	1.940	0.052405 .
Year2018:Hoverfly	-0.328587	0.186629	1.754	0.079406 .
Year2019:Hoverfly	-0.335724	0.183509	1.823	0.068293 .
Fly	0.015373	0.038627	0.397	0.691454
Honeybee	-0.014848	0.049707	0.297	0.766101
Year2018:Honeybee	-0.001064	0.044714	0.024	0.981132
Year2019:Honeybee	0.033299	0.099216	0.335	0.737587
Bumblebee	-0.003488	0.020740	0.167	0.867089

2017 (Year) is used as baseline levels for the models
Significance levels: . < 0.01, * <0.05, ** <0.01, ***<0.001.

Table S13. Linear mixed model average model output for fruit size deficits in response to abundance of different pollinator groups. All models within <2AIC of the global model averaged and full model-averaged coefficients shown.

Variable	Estimate	SE	z value	Pr(>|z|) d
Fly	0.0024838	0.0023393	1.058	0.290
Solitary bee	-0.0044053	0.0017224	2.542	0.011 *
Hoverfly	-0.0005791	0.0024875	0.232	0.817
Bumblebee	-0.0001621	0.0008487	0.190	0.849

Significance levels: . < 0.01, * <0.05, ** <0.01, ***<0.001.

Table S14. Linear mixed model average model output for seed set deficits in response to abundance of different pollinator groups. All models within <2AIC of the global model averaged and full model-averaged coefficients shown.

Variable	Estimate	SE	z value	Pr(>|z|) d
Bumblebee	-0.183213	0.056712	3.211	0.00132 **
Fly	0.061981	0.065763	0.940	0.34730
Hoverfly	0.020791	0.099801	0.208	0.83535
Honeybee	0.021869	0.004557	0.208	0.83556
Year2018	-0.008869	0.046033	0.192	0.84799
Year2019	-0.036901	0.063379	0.581	0.56141
Year2018:Hoverfly	-0.031964	0.094850	0.336	0.73692
Year2019:Hoverfly	-0.064635	0.150545	0.429	0.66803

2017 (Year) is used as baseline levels for the models
Significance levels: . < 0.01, * <0.05, ** <0.01, ***<0.001.

Table S15. Linear mixed model average model output for initial fruit set deficits in response to abundance of different pollinator groups. All models within <2AIC of the global model averaged and full model-averaged coefficients shown.

Variable	Estimate	SE	z value	Pr(>|z|) d
Year2018	-0.021339	0.052589	0.403	0.68679
Year2019	-0.151003	0.058039	2.587	0.00968 **
Bumblebee	-0.095913	0.042611	2.236	0.02532 *
Solitary bee	0.052011	0.051721	1.002	0.31619
Hoverfly	0.030068	0.041579	0.721	0.47095
Honeybee	-0.008682	0.024830	0.348	0.72758

2017 (Year) is used as baseline levels for the models
Significance levels: . < 0.01, * <0.05, ** <0.01, ***<0.001.

Table S16. Linear mixed model average model output for final fruit set deficits in response to abundance of different pollinator groups. All models within <2AIC of the global model averaged and full model-averaged coefficients shown.

Variable	Estimate	SE	z value	Pr(>|z|) d
Year2018	0.058998	0.061321	0.957	0.3386
Year2019	-0.125278	0.059987	2.077	0.0378 *
Hoverfly	0.139702	0.127815	1.091	0.2753
Year2018:Hoverfly	-0.074439	0.135146	0.550	0.5825
Year2019:Hoverfly	-0.077287	0.136707	0.564	0.5725
Fly	0.011489	0.031209	0.367	0.7138
Bumblebee	-0.002814	0.017043	0.164	0.8695
Honeybee	0.002374	0.015374	0.154	0.8779
Solitary bee	0.001737	0.015498	0.111	0.9113

2017 (Year) is used as baseline levels for the models
Significance levels: . < 0.01, * <0.05, ** <0.01, ***<0.001.
